# Selections from Favorite Prescriptions of Living American Practitioners

**Published:** 1859-03

**Authors:** 


ART. VII.—*Selections from Favorite Prescriptions of Living American Practitioners.* By HORACE GREEN, M. D., L.L. D., President of the Faculty, and Emeritus Professor of the Theory and Practice of Medicine in the New York Medical College; Corresponding Fellow of the London Medical Society; Member of the American Medical Association, etc. New York: WILEY & HALSTED, 351 Broadway. 1858.

A number of these prescriptions have already appeared in the pages of the *American Medical Monthly*, to which some have been added, and the whole is published in a neat octavo of two hundred pages, by Wiley & Halsted, of New York. The mechanical execution is admirable, and the work itself, though rather of a novel character, will undoubtedly be quite useful, as many of the formulæ are in constant use by our most eminent practitioners. There is, however, a tendency in it to lead to a routine, in place of a practice in accordance with the great principles of our science.

